# Mechanism and treatment of Sirtuin family in vascular calcification

**DOI:** 10.3389/fcvm.2025.1732281

**Published:** 2025-12-17

**Authors:** Zhexue Ren, Beibei Song, Yinying Peng, Ce Bian, Qi Shen, Mengjun Zhou, Bo Li, Xiaodong Jin

**Affiliations:** 1 School of Clinical Medicine, Shandong Second Medical University, Weifang, China; 2 Department of Cardiology, Zibo Central Hospital, Zibo, China; 3 Department of Geriatrics, Zibo Central Hospital, Zibo, China

**Keywords:** Sirtuin family, vascular calcification, vascular smooth muscle cells, inflammation, oxidative stress

## Abstract

With the acceleration of population aging, the prevalence of vascular calcification (VC) is on the rise, particularly among patients with hypertension, diabetes, chronic kidney disease, and age-related diseases. VC is characterized by the abnormal deposition of calcium phosphate in the vascular walls, and there are currently no effective pharmacological treatments available. This condition is a manifestation of vascular aging. The silent information regulator (SIRT) family, which includes SIRT1 to SIRT7, functions as deacetylases and plays a crucial role in cellular resistance, energy metabolism, apoptosis, and cellular aging, often referred to as longevity proteins. The SIRT family has shown potential in alleviating vascular aging by inhibiting inflammation, reducing endoplasmic reticulum stress, lowering mitochondrial oxidative stress, and promoting DNA damage repair, all of which contribute to the suppression of vascular calcification. Notably, SIRT1, SIRT2, SIRT3, SIRT6, and SIRT7 have demonstrated therapeutic potential in the treatment of vascular calcification.

## 1 Introduction

Vascular calcification (VC) encompasses a range of conditions characterized by the abnormal deposition of calcium and phosphorus within the vascular wall. It is increasingly acknowledged as an active and regulated process, akin to bone formation (1). The occurrence of VC involves the participation of various cell types, including macrophages, endothelial cells, and vascular smooth muscle cells (VSMCs), which play an essential role in this process (2). The types of calcification include intimal calcification, mesangial calcification, and valvular calcification (3). Among these, intimal calcification is associated with atherosclerosis (4). Medial calcification, also known as Mönckeberg sclerosis, preferentially occurs along the elastic lamina and is typically identified in the small and medium-sized arteries of the lower extremities. This condition is associated with advanced age, diabetes, and chronic kidney disease (CKD) (5, 6). Increasing evidence suggests a correlation between aging and VC (7). Several mechanisms contribute to VC, including inflammation, endoplasmic reticulum stress (ERS), mitochondrial dysfunction, ferroptosis, cell death, and DNA damage repair (8).

**Figure 1:**
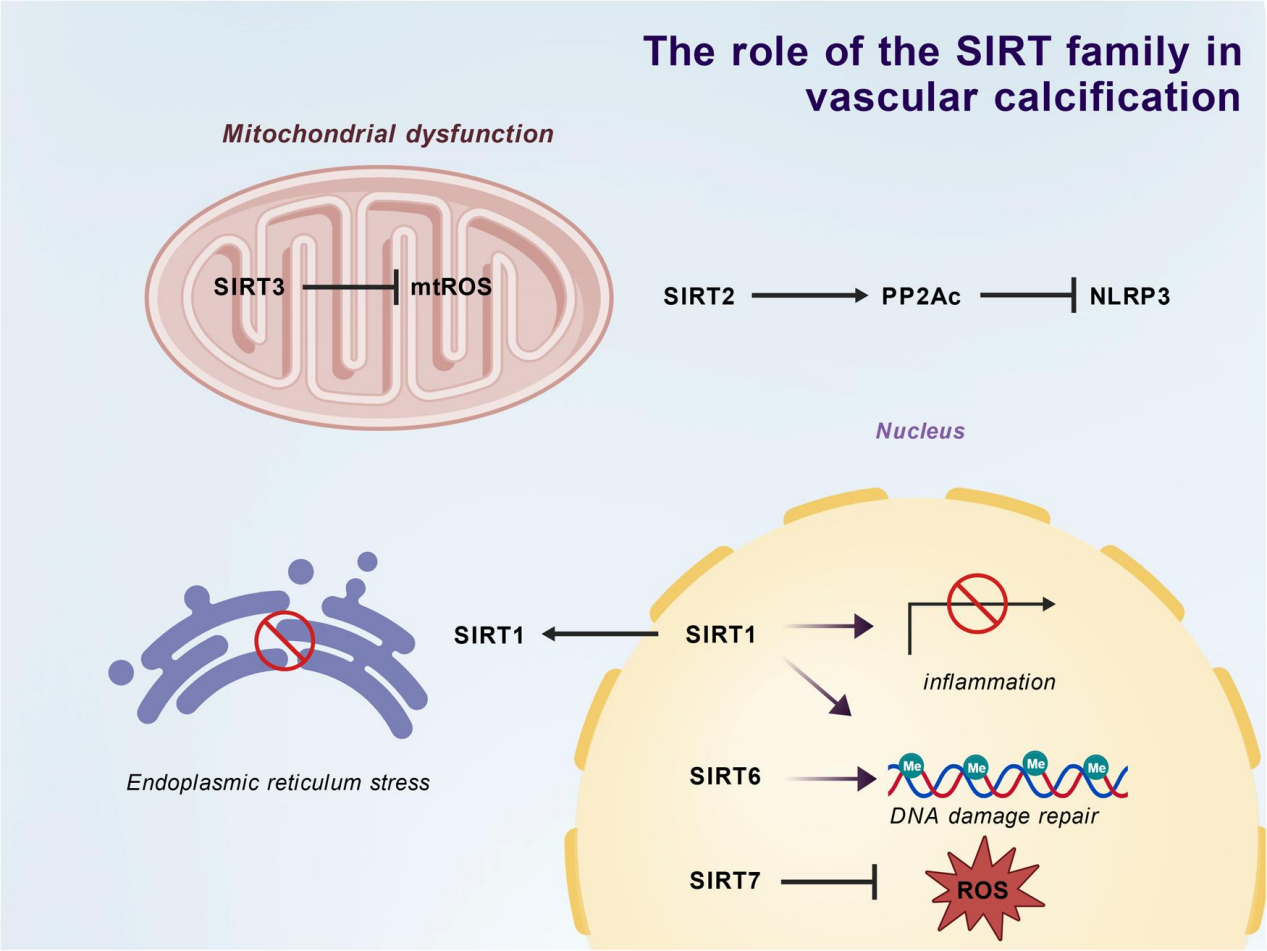
Diagram explaining the role of the SIRT family in VC. SIRT3 inhibits mtROS, mitigating mitochondrial dysfunction. SIRT2 activates PP2Ac, which inhibits NLRP3. SIRT1 reduces endoplasmic reticulum stress and inflammation in the nucleus and promotes DNA damage repair, while SIRT6 is involved in DNA damage repair. SIRT7 inhibits reactive oxygen species (ROS) within the nucleus.

The lysine deacetylase Sirtuin (SIRT) family of proteins comprises a widely distributed group of histone deacetylases found in various cell types. This extensive family consists of seven isoforms, namely SIRT1 to SIRT7 (9). SIRT proteins play crucial roles in cellular resistance, energy metabolism, apoptosis, and senescence (10). SIRT1 and SIRT2 are predominantly expressed in the nucleus and cytoplasm, while SIRT3, SIRT4, and SIRT5 are primarily localized in the mitochondria. SIRT6 and SIRT7 are located in the nucleus (11). Currently, SIRT1, SIRT2, SIRT3, SIRT6, and SIRT7 have been associated with VC. Among these, SIRT1, the most extensively studied isoform, inhibits VC primarily by suppressing endoplasmic reticulum stress (ERS) and promoting DNA damage repair (12, 13), SIRT3 inhibits VCmainly by reducing mitochondrial reactive oxygen species (mtROS) production (14), whereas SIRT6 mitigates VC by enhancing DNA damage repair (15, 16). Additionally, SIRT7 decreases calcification by reducing intracellular reactive oxygen species (ROS) accumulation and inhibiting nuclear factor erythroid 2-related factor 2 (Nrf2)-mediated oxidative stress (17, 18) (Figure 1).

## 2 Molecular mechanisms by which the SIRT family affects VC

Inflammation is a crucial immune response that occurs during infection or injury, playing a vital role in maintaining tissue homeostasis under various adverse conditions (19), Furthermore, there is a significant association between the occurrence of VC and inflammation (20). The upregulation of inflammatory cytokines, including interleukin (IL)-1β, IL-6, and tumor necrosis factor (TNF)-α, can activate downstream inflammatory pathways and facilitate disease progression (19). Elevated levels of IL-1β in medial arterial smooth muscle contribute to the induction of senescence-associated calcification (20). The IL-1β-induced cellular senescence is contingent upon the upregulation of BMP2, which is necessary for osteoclast transformation and subsequent calcification (21). Furthermore, evidence indicates that inflammatory responses can initiate and precede the osteogenic transformation of VSMCs (22). Nucleotide-binding domain, leucine-rich family-containing, pyrin domain-containing-3 (NLRP3) inflammatory vesicles play a crucial role in the calcification of VSMCs, and the inhibition of NLRP3 inflammatory vesicles has been shown to prevent VC (23). Naringin has been shown to inhibit the activation of NLRP3 inflammatory vesicles by promoting SIRT3-mediated mitophagy, thereby reducing NLRP3 inflammatory activity (24). Furthermore, chronic inflammation is recognized as a significant risk factor for aging and age-related diseases (25). NF-κB, a pivotal signaling molecule in inflammation, plays a critical role in both aging and VC. Elevated glucose and phosphate levels induce senescence and VC in VSMCs while concurrently inhibiting SIRT1 expression (26). SIRT1 can directly interact with NF-κB p65, leading to the deacetylation and subsequent inactivation of NF-κB, which inhibits both senescence and the osteogenic differentiation of VSMCs, thereby mitigating VC (26). Additionally, high phosphorus-induced calcification of VSMCs may be linked to premature cellular senescence and replicative senescence (27).

The endoplasmic reticulum (ER) serves as the primary site for protein synthesis and is a crucial reservoir of calcium ions (Ca^2+^). It plays a significant role in maintaining intracellular protein synthesis and stabilizing Ca^2+^ levels (28). ERS is a protective cellular response that mitigates the agglutination of unfolded proteins by reducing their concentration within the cell, thus preserving the intracellular Ca^2+^ balance. ERS exerts a protective effect by inducing the expression of endoplasmic reticulum molecular chaperones, such as glucose-regulated proteins GRP78 and GRP94 (29). Research has demonstrated that ERS can facilitate the osteogenic differentiation of VSMCs through three pathways: IRE1-XBP1, PERK-eIF2α-ATF4, and ATF6 (30). Additionally, spermidine has been shown to alleviate VC in CKD by upregulating SIRT1 and inhibiting the expression of ATF4 and CHOP in smooth muscle cells (12).

Mitochondria serve as central hubs for energy metabolism and play crucial roles in various biological processes. Abnormalities in mitochondrial function significantly influence the onset and progression of VC, with mitochondrial dysfunction identified as a key contributor to this condition (31). Furthermore, mitochondrial dysfunction and oxidative stress are recognized as critical causative factors and therapeutic targets in heart disease, kidney disease, and diabetes (32, 33). Oxidative stress acts as a significant mediator of VC, particularly in the context of medial calcification (34). Previous studies have demonstrated that the inactivation of SIRT3 results in hyperacetylation of superoxide dismutase 2 (SOD2), thereby exacerbating vascular oxidative stress (35). SIRT3 primarily operates within mitochondria, where elevated production of ROS triggers osteogenic transformation in VSMCs. It has been established that SIRT3 can reduce mitochondrial ROS (mtROS) levels by modulating the downstream regulator peroxisome proliferator-activated receptor-*γ* coactivator-1α (PGC-1α), which in turn decreases ROS levels and enhances the expression of SOD2 in VSMCs. This mechanism mitigates mtROS levels and inhibits abdominal aortic calcification and carotid arterial calcification *in vitro* under conditions of high phosphate-induced VC. The use of the SIRT3 inhibitor, 3-TYP, or the application of small interfering RNA (siRNA) to inhibit SIRT3 resulted in a reduction of PGC-1α-induced upregulation of both SOD1 and SOD2, thereby diminishing the protective effects of PGC-1α against calcification in VSMCs. Clinical investigations have revealed that PGC-1α levels are diminished in calcified femoral arteries of CKD patients. Additionally, in phosphate-induced calcification of human arteries, the upregulation of PGC-1α has been shown to inhibit the formation of calcium nodules, an effect that is negated by SIRT3 inhibitors (14).

Double-strand breaks (DSBs) represent the most severe form of DNA damage response, which is activated upon the occurrence of DSBs and leads to the activation of ataxia-telangiectasia mutated (ATM) protein. This activation results in the dissociation of ATM from its dimeric form, followed by self-phosphorylation at Ser1981, thereby facilitating the completion of DNA damage repair (36). In diabetic patients, the absence of SIRT1 accelerates VC induced by DNA damage. Studies have shown that SIRT1 expression is diminished while markers of DNA damage are elevated in calcified arteries of diabetic patients compared to non-diabetic and non-calcified controls. Furthermore, VSMCs isolated from diabetic patients exhibit increased DNA damage and cellular senescence. In the context of diabetes, the process of DNA damage-induced calcification is accelerated; however, *in vitro* activation of SIRT1 mitigates this calcification by enhancing the MRN repair complex within the vascular endothelium, highlighting its therapeutic potential for diabetic patients (13). Additionally, existing literature indicates that SIRT6 can inhibit the progression of VC in CKD patients by down-regulating RUNX2. Notably, the specific deletion of SIRT6 in VSMCs promotes VC through the inhibition of DNA damage repair (15, 37).

Insufficient levels of SIRT6 lead to the accumulation of DNA damage, which activates the DNA damage response pathway. This activation subsequently results in the phosphorylation of NF-κB through ATM/ATR kinases, indirectly promoting inflammation, a phenomenon referred to as “inflammaging.” Conversely, SIRT1 inhibits NF-κB, thereby reducing inflammation-mediated oxidative stress, lowering the risk of DNA damage, and indirectly supporting the repair functions of SIRT6. SIRT1 primarily addresses acute stress responses, such as inflammation inhibition, and maintains short-term cellular homeostasis by regulating transcription factors like NF-κB. In contrast, SIRT6 concentrates on long-term genomic maintenance, including DNA repair, and delays the aging process through chromatin regulation. Together, these two proteins form a network via the stress signaling pathway (NF-κB) to collectively counteract the cascade of inflammation, aging, and DNA damage.

**Figure 2:**
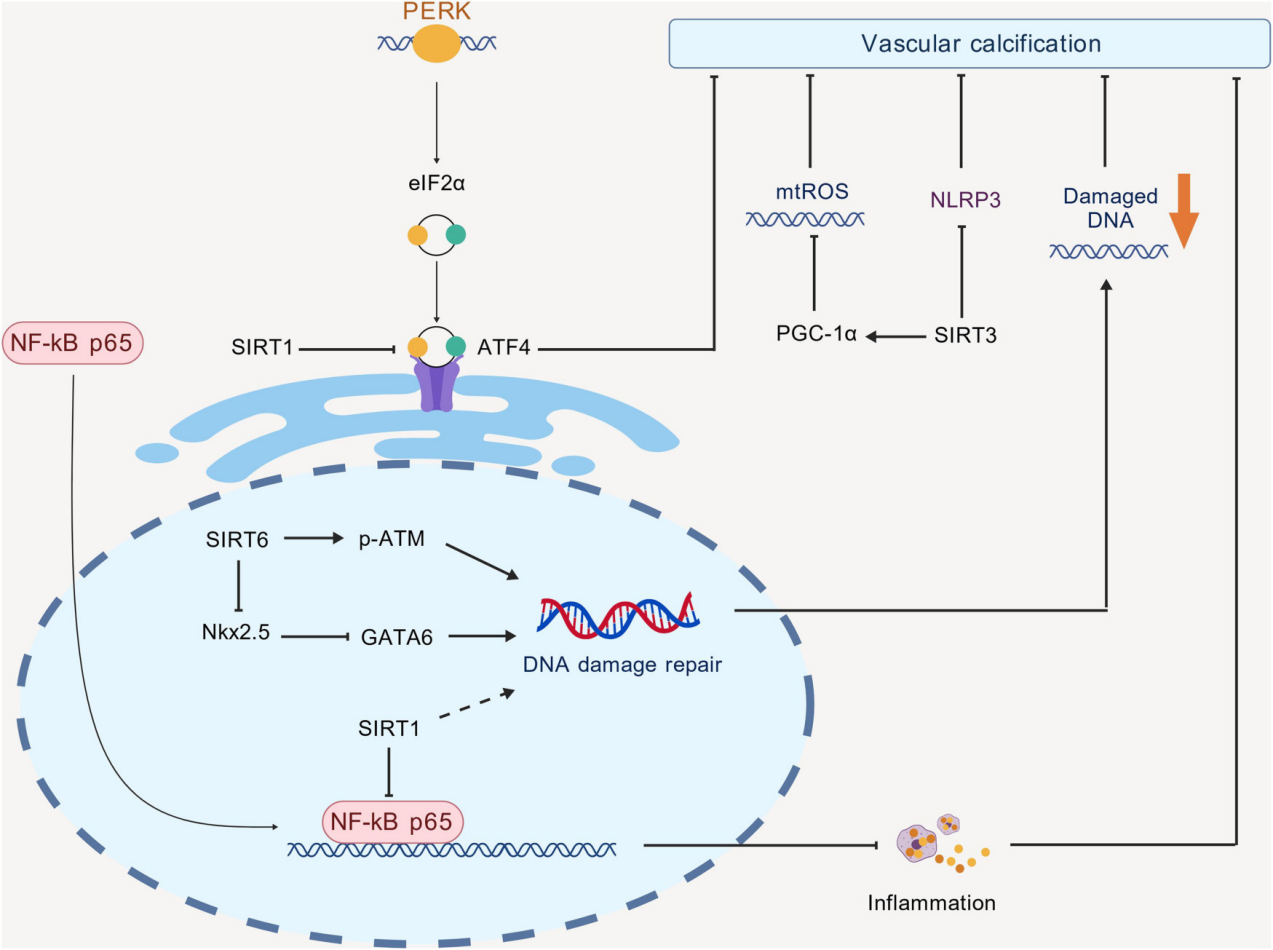
Mechanisms by which the SIRT family affects VC. The PERK-eIF2α-ATF4 pathway is a critical pathway involved in ERS. SIRT1 inhibits VC by suppressing ATF4. Additionally, SIRT1 can bind to NF-κB p65 in the nucleus to inhibit inflammatory responses and also participates in DNA damage repair, further suppressing VC. SIRT6 inhibits VC by promoting p-ATM and downregulating Nkx2.5, which in turn downregulates GATA6 to enhance DNA damage repair. SIRT3 promotes PGC-1α, inhibits mtROS, and suppresses NLRP3 to prevent VC.

Clinical studies have demonstrated that the expression of SIRT6 is significantly lower in patients with CKD compared to healthy individuals, and this expression is negatively correlated with the thoracic aortic VC Agatston score (37). Recent findings indicate that GATA6 contributes to the development of VC by accelerating aging-related arterial calcification in VSMCs, which occurs through the inhibition of SIRT6 activity and the obstruction of DNA damage repair mechanisms. The accumulation of DNA damage is a crucial factor in both aging and osteogenic differentiation within VSMCs. During vascular calcification, SIRT6 expression is diminished, and the knockdown of SIRT6 exacerbates DNA damage during the calcification process in VSMCs, as evidenced by an increase in the expression of *γ*-H2AX, a biomarker for DNA double-strand breaks. The administration of the DNA repair agent AV-153 effectively repaired DNA damage and mitigated the adverse effects associated with SIRT6 knockdown. Furthermore, SIRT6 positively regulates the expression of phosphorylated ATM (p-ATM), and its function is contingent upon ATM activation for the repair of DNA double-strand breaks and the inhibition of vascular calcification. Additionally, Nkx2.5 enhances GATA6 transcription, while SIRT6 inhibits GATA6 transcription through deacetylation and the promotion of Nkx2.5 degradation (16) (Figure 2).

## 3 Application of SIRT family in VC

Currently, among the SIRT family, SIRT1, SIRT3, SIRT6, and SIRT7 have been extensively studied, with SIRT1 being the most well-researched. Notably, SIRT2 is the only member predominantly located in the cytoplasm. Recent studies have demonstrated that colchicine inhibits VC by enhancing the SIRT2-PP2Ac signaling pathway, thereby reducing the activation of NLRP3 (38). This discovery identifies SIRT2 as a novel target for the treatment of VC.

Furthermore, there are currently no relevant reports on the roles of SIRT4 and SIRT5 inVC, although SIRT4 and SIRT5 are primarily located in the mitochondria. SIRT4 plays a critical role in aging-related diseases, primarily by regulating mitochondrial metabolism, inhibiting inflammatory responses, and modulating cell apoptosis and cellular stress responses through its ADP-ribosyltransferase and deacetylase activities (39). SIRT4 may improve VC by regulating oxidative stress and inflammatory responses. It is proposed that SIRT4 reduces oxidative damage in VSMCs by downregulating the activity of mtROS-generating enzymes, such as NADPH oxidase. Additionally, SIRT4 can inhibit the activation of the NF-κB pathway, thereby reducing the release of inflammatory factors, such as TNF-α and IL-6, and delaying the formation of a calcified microenvironment.

SIRT5 exhibits limited deglycase activity and functions as a desuccinylase, demalonylase, and deglutaminase. It primarily regulates protein substrates involved in various metabolic processes, including glycolysis, the tricarboxylic acid cycle, and the urea cycle (40). SIRT5 can modulate key enzymes in the tricarboxylic acid cycle, such as citrate synthase and succinate dehydrogenase, through desuccinylation modifications, thereby maintaining the balance of cellular energy metabolism. During the process of VC, SIRT5 may diminish the osteogenic differentiation tendency of VSMCs by inhibiting glycolysis and enhancing oxidative phosphorylation.

Members of the SIRT family play significant roles in VC. The existing research gaps should not be interpreted as a lack of importance. Through the cross-integration of metabolomics and vascular biology, the functions of SIRT4 and SIRT5 may present new breakthroughs in the future study of the mechanisms underlying VC.

### 3.1 Application of SIRT1 in VC

The natural compounds resveratrol, intermediate protein 1–53, terpinen-4-ol, and spermidine (Spd) have been demonstrated to inhibit VC through the modulation of SIRT1 activity (12, 41–43).

The drugs utilized in clinical practice include Compound Danshen Dripping Pills (CDDP), luteolin, and the SGLT2 inhibitor dapagliflozin (DAPA). Recent studies have demonstrated that CDDP inhibits the expression markers associated with VC by suppressing the Wnt/β-catenin pathway through the upregulation of the upstream inhibitor of Wnt, DKK1-LRP6. Additionally, CDDP activates SIRT1, which reduces the expression markers of senescence, including p21, p16, and SA-β-gal, thereby exerting an anti-vascular senescence effect that subsequently inhibits the occurrence of intimal calcification in atherosclerotic lesions (44). Luteolin mitigates VC by reducing oxidative stress and enhancing autophagy levels, through modulation of the SIRT1/CXCR4 signaling pathway (45). DAPA exerts its anti-calcification effects on VSMCs by directly targeting SGLT2, with the overexpression of SGLT2 being sufficient to diminish these beneficial effects. Furthermore, DAPA effectively limits glucose levels and the NAD+/NADH ratio in calcified VSMCs, upregulating SIRT1 in a caloric restriction-dependent manner (46). The DAPA/SGLT2/SIRT1 axis provides novel insights into the potential of SGLT2 inhibitors in preventing and treating VC.

In addition, certain target genes can be leveraged to inhibit the progression of VC. Circular RNAs (circRNAs) represent a class of non-coding RNAs characterized by a closed-loop structure. Notably, circHIPK3 has been shown to alleviate VC via the FUS/SIRT1/PGC-1α/MFN2 axis (47). Furthermore, senescence-associated miR-34a has been implicated in promoting VC through the calcification of VSMCs by directly down-regulating Axl and SIRT1, which inhibits cell proliferation and induces senescence, respectively. Consequently, the inhibition of miR-34a presents a promising therapeutic strategy for VC treatment (48). Additionally, it has been demonstrated that the overexpression of HOTAIR mitigates Pi-induced calcification by modulating the miR-126/Klotho/SIRT1 axis, thereby inhibiting the Wnt/β-catenin signaling pathway (49). This finding suggests a novel potential target gene for the clinical management of VC.

Adenosine-activated AMPK has been shown to inhibit the expression of H19 and Runx2 in SAH hydrolase-deficient VSMCs by inducing SIRT1-mediated histone H3 hypoacetylation and DNMT3b-mediated hypermethylation of the H19 promoter. This process subsequently inhibits osteogenic differentiation in VSMCs (50). These findings suggest that non-coding RNAs (such as circRNA and miRNA) and associated regulatory axes (including SIRT1 and AMPK) represent potential molecular targets for the prevention and treatment of VC.

Recent studies have demonstrated that the overexpression of SIRT1 enhances the expression of its downstream targets, PGC-1α and Mfn2. Additionally, it reduces the levels of β-galactosidase and ROS, inhibits apoptosis in VSMCs, and increases ATP secretion (51). It provides potential molecular targets for the prevention and treatment of arterial diseases in elderly patients.

SIRT1-targeted drugs have demonstrated a significant anti-VC effect in experimental models. However, three major challenges must be addressed for successful clinical translation: the development of a vascular-targeted delivery system to enhance efficacy and minimize systemic toxicity, the establishment of biomarkers for SIRT1 pathway activity to facilitate precise treatment, and the design of clinical trials focused on definitive endpoints related to vascular calcification. Future research should integrate multi-omics technologies to analyze the regulatory network of SIRT1 within the complex pathological microenvironment and investigate synergistic strategies with other anti-calcification targets, such as SIRT6, ultimately advancing the translation of SIRT1 activators from the laboratory to clinical practice.

### 3.2 Application of SIRT3 in VC

SIRT3 has emerged as a promising therapeutic target for ameliorating VC (14). Compounds such as IMD, Nε-carboxymethyl lysine (CML), and extracts from Begonia alba (ECE) have been shown to inhibit VC through the modulation of SIRT3.

Research indicates that IMD enhances mitochondrial function and reduces mtROS by up-regulating SIRT3; this effect can be blocked by pretreatment with the SIRT3 inhibitor 3-TYP. Furthermore, IMD mitigates VC by improving mitochondrial function and reducing mitochondrial oxidative stress in CKD through the up-regulation of SIRT3 (52).

CML was found to significantly enhance the expression and nuclear translocation of nuclear factor 1 of activated T cells (NFATc1) in VSMCs and the mouse aorta. CML increases the acetylation of NFATc1 at the K549 site by inhibiting the deacetylase SIRT3 and antagonizing focal adhesion kinase (FAK). This action reduces the phosphorylation of NFATc1 at the Y270 site, facilitating crosstalk between acetylation and phosphorylation. Consequently, this process affects the nuclear translocation of NFATc1, ultimately contributing to the development of VC in diabetic patients (53).

ECE has been shown to mitigate hypertension-associated VC by upregulating PGC-1α and SIRT3, enhancing SOD2 activity, decreasing mitochondrial DNA damage, and reducing mtROS levels (54).

SIRT3 demonstrates potential protective effects against VC through the regulation of mitochondrial function, oxidative stress, and inflammatory responses. However, the cell specificity of its mechanisms, dependence on disease stages, and barriers to clinical translation require further in-depth exploration.

### 3.3 Application of SIRT6 in VC

It was observed that the up-regulation of SIRT6 inhibits VC, while the knockdown of SIRT6 results in pronounced VC in CKD. SIRT6 can deacetylate RUNX2, thereby promoting its ubiquitin-proteasome degradation through exportin 1-dependent nuclear export. This process leads to the down-regulation of RUNX2, a reduction in the osteogenic differentiation of VSMCs, and ultimately inhibits VC, positioning SIRT6 as a potential target for clinical intervention in VC (37).

Bone marrow mesenchymal stem cell-derived exosomes have been shown to attenuate renal fibrosis and inflammation. Elevated levels of aortic calcification promote VC in CKD through the Wnt/β-catenin signaling pathway. SIRT6 regulates the cytoplasmic lysosomal transport of serum high mobility group 1 (HMGB1) and inhibits the expression of β-catenin target genes via deacetylation, thereby preventing fibrosis in CKD. Furthermore, BMSC-derived exosomes inhibit hyperphosphate-induced aortic calcification and enhance renal function through the SIRT6-HMGB1 deacetylation pathway (55). SIRT6 plays an important role in enhancing renal function and preventing aortic calcification.

A high-fat diet (HFD) and palmitic acid have been shown to promote calcification and decrease SIRT6 expression in the aorta and VSMCs, respectively. Palmitic acid induces apoptosis in smooth muscle cells, with significant increases in the molecular apoptotic markers Cleaved-Caspase3 and pro-apoptotic BAX observed in VSMCs treated with palmitic acid. However, the overexpression of SIRT6 ameliorates the calcification and apoptosis induced by palmitic acid. Furthermore, SIRT6 overexpression significantly mitigates the palmitate- and phosphate-induced elevation of BMP2 and RUNX2 in VSMCs. These findings suggest that saturated fatty acids promote calcification by inhibiting SIRT6 expression in vascular smooth muscle cells (56). Therefore, SIRT6 has a protective effect on palmitic acid-induced VC.

Capsaicin has been found to inhibit osteoblast transdifferentiation by activating the transient receptor potential vanilloid type 1 (TRPV1). This activation increases the expression of SIRT6, which promotes the deacetylation and degradation of hypoxia-inducible factor-1α (HIF-1α) via the proteasome. Consequently, capsaicin slows down atherosclerotic calcification by enhancing SIRT6-mediated deacetylation and degradation of HIF-1α. Furthermore, clinical research has demonstrated that the risk of coronary artery calcification (CAC) is lower in groups that consume capsaicin. Specifically, CAC scores decrease with increasing daily chili pepper consumption, indicating that chili pepper intake may have the potential to prevent CAC (57). In addition, activation of HIF-1α is associated with ERS (58). Future studies should investigate whether capsaicin can inhibit the activation of HIF-1α by alleviating ERS, thereby preventing the occurrence of VC.

The administration of liraglutide (LRGT) has been shown to improve medial VC and significantly mitigate age-related increases in both systolic blood pressure (SBP) and diastolic blood pressure (DBP), as well as the senescence-associated proteins p53 and p16, and the inflammatory cytokines TNF-α and IL-6. Furthermore, LRGT treatment resulted in a decrease in malondialdehyde (MDA), 8-hydroxydeoxyguanosine (8-OHdG), and Keap1 levels, while increasing glutathione (GSH), Nrf2, and its target antioxidants, including heme oxygenase-1 (HO-1), NAD(P)H dehydrogenase [quinone] 1 (NQO1), and glutamate-cysteine ligase catalytic subunit (GCLC). Additionally, LRGT enhanced the immune expression of endothelial nitric oxide synthase (eNOS) in aged rats. At the molecular level, LRGT was found to upregulate the mRNA expression of SIRT6 in the aorta and downregulate the transcriptional levels of its upstream microRNA, MiR-34a (59). In conclusion, LRGT mitigates medial arterial calcification associated with physiological aging by inhibiting cellular senescence through the MiR-34a/SIRT6 pathway and restoring the Keap1/Nrf2 antioxidant cascade.

Panaxynol (PA) inhibits soluble epoxide hydrolase (sEH) by activating SIRT6, which increases the levels of the lipid signaling molecule 14,15-EET. PA preserves vascular structure and function by reducing the infiltration of inflammatory macrophages in perivascular adipose tissue and alleviates diabetes-induced VC (60). Consequently, PA has emerged as a potential natural therapeutic strategy for mitigating diabetic vascular complications.

SIRT6 demonstrates a significant protective effect against VC by targeting Runx2, HIF-1α, and senescence-related pathways. Investigating its mechanisms offers new targets for the prevention and treatment of high-risk populations, such as individuals with CKD. Nevertheless, limitations in disease models, the impact of cellular heterogeneity, and the safety of intervention methods remain critical challenges that hinder clinical translation. In the future, integrating multi-omics technologies will be essential for analyzing the regulatory network of SIRT6 within the complex pathological microenvironment. Additionally, developing more targeted intervention strategies is crucial for successfully transitioning from basic research to clinical application.

### 3.4 Application of SIRT7 in VC

Nrf2 is a master transcription factor that regulates cellular redox homeostasis by activating antioxidant response element (ARE)-responsive genes (61). Hesperidin has been shown to enhance Nrf2 expression through the upregulation of SIRT7, which subsequently activates the ARE, inhibits lipopolysaccharide-induced NF-κB inflammatory cytokine secretion and osteogenic factor expression, and reduces ROS production and apoptosis. This suggests that hesperidin may be beneficial in the prevention of calcific aortic valve disease and warrants further exploration for its potential application in VC treatment (17).

Clinical cross-sectional studies have demonstrated that coronary artery calcification in patients with type 2 diabetes undergoing coronary angiography correlates with elevated levels of myeloid calcifying cells in monocytes and increased expression of RUNX2. Knockdown of SIRT7 led to a decrease in RUNX2 deacetylation and an increase in VC. Furthermore, hyperglycemia promotes coronary artery calcification in diabetes by inducing miR-125b-5p through the JAK/STAT signaling pathway, which subsequently reduces SIRT7 expression in the human myeloid cell line THP-1 (62).

SIRT7 has been shown to reduce intracellular ROS accumulation and inhibit Nrf2-mediated oxidative stress. Furthermore, SIRT7 accelerates cell cycle progression, thereby delaying cellular senescence and contributing to the prevention and control of VC development (18).

Recent studies have demonstrated that Ganoderma lucidum spore powder (GLSP) downregulates the expression of cell cycle regulatory genes P16 and P21, as well as the senescence-associated secretory phenotype (SASP) factors IL-1β, TNF-α, MMP3, MMP13, ICAM-1, and VCAM-1. Additionally, GLSP reduces the levels of mtROS and downregulates the expression of DNA damage-related proteins *γ*H2AX, p-Chk1, and autophagy-related proteins P62 and LC3. Furthermore, GLSP upregulates the expression of SIRT7, which promotes the deacetylation of Keap1, facilitating the dissociation of the Keap1-Nrf2 complex and enhancing the nuclear translocation and activation of Nrf2 (63). In conclusion, GLSP exerts an anti—vascular aging effect by regulating the cell cycle and SASP, alleviating DNA damage, reducing oxidative stress, improving mitochondrial function and regulating metabolic levels. GLSP improves atherosclerosis and VC associated with vascular aging *in vivo*. It was confirmed that SIRT7 could be a target for inhibiting VC development.

SIRT7 demonstrates a significant protective effect against VC by modulating oxidative stress and cellular senescence pathways. Its unique targeting potential is particularly noteworthy in the context of calcification associated with CKD. At present, relevant pharmacological agents remain in the early stages of research and development. In practical applications, a comprehensive evaluation should be conducted, taking into account the patient's underlying conditions and the expression levels of SIRT7.

## 4 Conclusion and outlook

The occurrence of VC involves the participation of multiple factors, primarily attributed to the abnormal deposition of calcium and phosphorus in the vascular wall. This article primarily discusses how the SIRT family can ameliorate VC through various mechanisms, including inflammation, ERS, mitochondrial dysfunction, and DNA damage repair. Recently, some studies have confirmed that ferroptosis can promote VC (64), indicating that metformin may alleviate the development of hyperlipidemia-associated VC by inhibiting ferroptosis (65). Ferroptosis is a form of cell death characterized by iron-dependent lipid peroxidation, regulated by multiple pathways, including redox balance, lipid metabolism, and energy metabolism (66). Metformin enhances autophagy and inhibits abnormal cell proliferation through the AMPK/SIRT1-FoxO1 pathway, thereby mitigating oxidative stress in diabetic nephropathy (67). Previous studies have demonstrated that metformin can increase the expression of SIRT3 and GPX4, significantly elevate the levels of p-mTOR and p-AMPK, and improve polycystic ovary syndrome in mice by inhibiting ovarian ferroptosis (68).

SIRT proteins may serve as crucial intermediates for metformin's inhibition of ferroptosis-related vascular calcification. They play a synergistic role by regulating the antioxidant system, iron metabolism, and cellular phenotype transformation. Future research should concentrate on specific activation strategies for SIRT proteins, such as selective agonists, to enhance the targeted therapeutic effects of metformin.

Hesperidin has been shown to prevent the development of calcific aortic valve disease via the SIRT7-Nrf2-ARE axis (17). Future studies could further investigate the SIRT family's pathways that inhibit VC through ferroptosis. Moreover, the SIRT family influences VC through various signaling pathways, including the Wnt/β-catenin, Runx2, NF-κB, and JAK/STAT pathways, as well as the AMPK signaling pathway. Additionally, the role of the SIRT family in VC is noteworthy, with current research primarily focusing on SIRT1, SIRT2, SIRT3, SIRT6, and SIRT7, while the functions of other SIRT proteins in VC remain to be explored. Clinically, it has been observed that a significant number of patients requiring coronary intervention exhibit multiple calcifications in the vessel walls; thus, investigating methods to prevent and delay the progression of VC is a promising area for future research.

## References

[1] Chen Y Zhao X Wu H. Arterial stiffness: a focus on vascular calcification and its link to bone mineralization. Arterioscler Thromb Vasc Biol. (2020) 40(5):1078–93. 10.1161/ATVBAHA.120.313131 32237904 PMC7199843

[2] Leopold JA. Vascular calcification: mechanisms of vascular smooth muscle cell calcification. Trends Cardiovasc Med. (2015) 25(4):267–74. 10.1016/j.tcm.2014.10.021 25435520 PMC4414672

[3] Kang J-H Kawano T Murata M Toita R. Vascular calcification and cellular signaling pathways as potential therapeutic targets. Life Sci. (2024) 336:122309. 10.1016/j.lfs.2023.122309 38042282

[4] Ortega MA De Leon-Oliva D Gimeno-Longas MJ Boaru DL Fraile-Martinez O García-Montero C Vascular calcification: molecular networking, pathological implications and translational opportunities. Biomolecules. (2024) 14(3):275. 10.3390/biom14030275 38540696 PMC10968665

[5] Lanzer P Hannan FM Lanzer JD Janzen J Raggi P Furniss D Medial arterial calcification. J Am Coll Cardiol. (2021) 78(11):1145–65. 10.1016/j.jacc.2021.06.049 34503684 PMC8439554

[6] Kim TI Guzman RJ. Medial artery calcification in peripheral artery disease. Front Cardiovasc Med. (2023) 10:1093355. 10.3389/fcvm.2023.1093355 36776265 PMC9909396

[7] Pescatore LA Gamarra LF Liberman M. Multifaceted mechanisms of vascular calcification in aging. Arterioscler Thromb Vasc Biol. (2019) 39(7):1307–16. 10.1161/ATVBAHA.118.311576 31144990

[8] Pan W Jie W Huang H. Vascular calcification: molecular mechanisms and therapeutic interventions. Medcomm. (2023) 4(1):e200. 10.1002/mco2.200 36620697 PMC9811665

[9] North BJ Verdin E. Sirtuins: sir2-related NAD-dependent protein deacetylases. Genome Biol. (2004) 5(5):224. 10.1186/gb-2004-5-5-224 15128440 PMC416462

[10] Alhazzazi TY Kamarajan P Verdin E Kapila YL. SIRT3 And cancer: tumor promoter or suppressor? Biochim Biophys Acta Rev Cancer. (2011) 1816(1):80–8. 10.1016/j.bbcan.2011.04.004 PMC3129516 21586315

[11] Michishita E Park JY Burneskis JM Barrett JC Horikawa I. Evolutionarily conserved and nonconserved cellular localizations and functions of human SIRT proteins. Mol Biol Cell. (2005) 16(10):4623–35. 10.1091/mbc.e05-01-0033 16079181 PMC1237069

[12] Liu X Chen A Liang Q Yang X Dong Q Fu M Spermidine inhibits vascular calcification in chronic kidney disease through modulation of SIRT1 signaling pathway. Aging Cell. (2021) 20(6):e13377. 10.1111/acel.13377 33969611 PMC8208796

[13] Bartoli-Leonard F Wilkinson FL Schiro A Serracino Inglott F Alexander MY Weston R. Loss of SIRT1 in diabetes accelerates DNA damage-induced vascular calcification. Cardiovasc Res. (2021) 117(3):836–49. 10.1093/cvr/cvaa134 32402066 PMC7898956

[14] Feng H Wang J-Y Yu B Cong X Zhang W-G Li L Peroxisome proliferator-activated receptor-*γ* coactivator-1α inhibits vascular calcification through sirtuin 3-mediated reduction of mitochondrial oxidative stress. Antioxid Redox Signaling. (2019) 31(1):75–91. 10.1089/ars.2018.7620 30829051

[15] Wang S Li L Liang Q Ye Y Lan Z Dong Q Deletion of SIRT6 in vascular smooth muscle cells facilitates vascular calcification via suppression of DNA damage repair. J Mol Cell Cardiol. (2022) 173:154–68. 10.1016/j.yjmcc.2022.10.009 36367517

[16] Li X Liu A Xie C Chen Y Zeng K Xie C The transcription factor GATA6 accelerates vascular smooth muscle cell senescence-related arterial calcification by counteracting the role of anti-aging factor SIRT6 and impeding DNA damage repair. Kidney Int. (2024) 105(1):115–31. 10.1016/j.kint.2023.09.028 37914087

[17] Zhao H Xian G Zeng J Zhong G An D Peng Y Hesperetin, a promising dietary supplement for preventing the development of calcific aortic valve disease. Antioxidants. (2022) 11(11):2093. 10.3390/antiox11112093 36358465 PMC9687039

[18] Yu H Xie Y Lan L Ma S Mok SWF Wong IN Sirt7 Protects against vascular calcification via modulation of reactive oxygen species and senescence of vascular smooth muscle cells. Free Radical Biol Med. (2024) 223:30–41. 10.1016/j.freeradbiomed.2024.07.021 39053861

[19] Medzhitov R. Inflammation 2010: new adventures of an old flame. Cell. (2010) 140(6):771–6. 10.1016/j.cell.2010.03.006 20303867

[20] Cai X Tintut Y Demer LL. A potential new link between inflammation and vascular calcification. J Am Heart Assoc. (2023) 12(1):e028358. 10.1161/JAHA.122.028358 36537336 PMC9973574

[21] Han L Zhang Y Zhang M Guo L Wang J Zeng F Interleukin-1β-induced senescence promotes osteoblastic transition of vascular smooth muscle cells. Kidney Blood Press Res. (2020) 45(2):314–30. 10.1159/000504298 32126555

[22] Viegas C Araújo N Marreiros C Simes D. The interplay between mineral metabolism, vascular calcification and inflammation in chronic kidney disease (CKD): challenging old concepts with new facts. Aging. (2019) 11(12):4274–99. 10.18632/aging.102046 31241466 PMC6628989

[23] u C Zhang C Kuang Z Zheng Q. The role of NLRP3 inflammasome activities in bone diseases and vascular calcification. Inflammation. (2021) 44(2):434–49. 10.1007/s10753-020-01357-z 33215255 PMC7985100

[24] Wang J Jing X Liu X Chen F Ge Z Liu X Naringin safeguards vertebral endplate chondrocytes from apoptosis and NLRP3 inflammasome activation through SIRT3-mediated mitophagy. Int Immunopharmacol. (2024) 140:112801. 10.1016/j.intimp.2024.112801 39121608

[25] Chung HY Cesari M Anton S Marzetti E Giovannini S Seo AY Molecular inflammation: underpinnings of aging and age-related diseases. Ageing Res Rev. (2009) 8(1):18–30. 10.1016/j.arr.2008.07.002 18692159 PMC3782993

[26] Zhang M Li T Tu Z Zhang Y Wang X Zang D Both high glucose and phosphate overload promote senescence-associated calcification of vascular muscle cells. Int Urol Nephrol. (2022) 54(10):2719–31. 10.1007/s11255-022-03195-4 35396645

[27] Takemura A Iijima K Ota H Son B-K Ito Y Ogawa S Sirtuin 1 retards hyperphosphatemia-induced calcification of vascular smooth muscle cells. Arterioscler Thromb Vasc Biol. (2011) 31(9):2054–62. 10.1161/ATVBAHA.110.216739 21719763

[28] Groenendyk J Agellon LB Michalak M. Calcium signaling and endoplasmic reticulum stress. Int Rev Cell Mol Biol. (2021) 363:1–20. 10.1016/bs.ircmb.2021.03.003 34392927

[29] Furmanik M van Gorp R Whitehead M Ahmad S Bordoloi J Kapustin A Endoplasmic reticulum stress mediates vascular smooth muscle cell calcification via increased release of Grp78 (glucose-regulated protein, 78 kDa)-loaded extracellular vesicles. Arterioscler Thromb Vasc Biol. (2021) 41(2):898–914. 10.1161/ATVBAHA.120.315506 33297752 PMC7837691

[30] Rao Z Zheng Y Xu L Wang Z Zhou Y Chen M Endoplasmic reticulum stress and pathogenesis of vascular calcification. Front Cardiovasc Med. (2022) 9:918056. 10.3389/fcvm.2022.918056 35783850 PMC9243238

[31] Zeng ZL Yuan Q Zu X Liu J. Insights into the role of mitochondria in vascular calcification. Front Cardiovasc Med. (2022) 9:879752. 10.3389/fcvm.2022.879752 35571215 PMC9099050

[32] Peoples JN Saraf A Ghazal N Pham TT Kwong JQ. Mitochondrial dysfunction and oxidative stress in heart disease. Exp Mol Med. (2019) 51(12):1–13. 10.1038/s12276-019-0355-7 31857574 PMC6923355

[33] Zhang Z Huang Q Zhao D Lian F Li X Qi W. The impact of oxidative stress-induced mitochondrial dysfunction on diabetic microvascular complications. Front Endocrinol (Lausanne). (2023) 14:1112363. 10.3389/fendo.2023.1112363 36824356 PMC9941188

[34] Agharazii M St-Louis R Gautier-Bastien A Ung R-V Mokas S Larivière R Inflammatory cytokines and reactive oxygen species as mediators of chronic kidney disease-related vascular calcification. Am J Hypertens. (2015) 28(6):746–55. 10.1093/ajh/hpu225 25430697

[35] Dikalova AE Itani HA Nazarewicz RR McMaster WG Flynn CR Uzhachenko R Sirt3 Impairment and SOD2 hyperacetylation in vascular oxidative stress and hypertension. Circ Res. (2017) 121(5):564–74. 10.1161/CIRCRESAHA.117.310933 28684630 PMC5562527

[36] Marechal A Zou L. DNA Damage sensing by the ATM and ATR kinases. Cold Spring Harbor Perspect Biol. (2013) 5(9):a012716. 10.1101/cshperspect.a012716 PMC3753707 24003211

[37] Li W Feng W Su X Luo D Li Z Zhou Y SIRT6 Protects vascular smooth muscle cells from osteogenic transdifferentiation via Runx2 in chronic kidney disease. J Clin Invest. (2022) 132(1):e150051. 10.1172/JCI150051 34793336 PMC8718147

[38] Yang S Huang H Jiang K Peng Y Liang Z Gong X Colchicine inhibits vascular calcification by suppressing inflammasome activation through the enhancement of the Sirt2-PP2Ac signaling pathway. J Biol Chem. (2025) 301(7):110381. 10.1016/j.jbc.2025.110381 40523615 PMC12274824

[39] He L Liu Q Cheng J Cao M Zhang S Wan X SIRT4 In ageing. Biogerontology. (2023) 24(3):347–62. 10.1007/s10522-023-10022-5 37067687

[40] Wang Y Chen H Zha X. Overview of SIRT5 as a potential therapeutic target: structure, function and inhibitors. Eur J Med Chem. (2022) 236:114363. 10.1016/j.ejmech.2022.114363 35436671

[41] Zhang P Li Y Du Y Li G Wang L Zhou F. Resveratrol ameliorated vascular calcification by regulating Sirt-1 and Nrf2. Transplant Proc. (2016) 48(10):3378–86. 10.1016/j.transproceed.2016.10.023 27931585

[42] Chen Y Zhang L-S Ren J-L Zhang Y-R Wu N Jia M-Z Intermedin1-53 attenuates aging-associated vascular calcification in rats by upregulating Sirtuin 1. Aging. (2020) 12(7):5651–74. 10.18632/aging.102934 32229709 PMC7185112

[43] Zhang Y He L Tu M Huang M Chen Y Pan D The ameliorative effect of terpinen-4-ol on ER stress-induced vascular calcification depends on SIRT1-mediated regulation of PERK acetylation. Pharmacol Res. (2021) 170:105629. 10.1016/j.phrs.2021.105629 34089864

[44] Yang Y Yuan L Xiong H Guo K Zhang M Yan T Inhibition of vascular calcification by compound danshen dripping pill through multiple mechanisms. Phytomedicine. (2024) 129:155618. 10.1016/j.phymed.2024.155618 38678949

[45] Yu X Xu L Su C Wang C Wang Z Wang Y Luteolin protects against vascular calcification by modulating SIRT1/CXCR4 signaling pathway and promoting autophagy. AAPS J. (2024) 26(6):111. 10.1208/s12248-024-00982-y 39438407

[46] Li L Liu H Chai Q Wei J Qin Y Yang J Dapagliflozin targets SGLT2/SIRT1 signaling to attenuate the osteogenic transdifferentiation of vascular smooth muscle cells. Cell Mol Life Sci. (2024) 81(1):448. 10.1007/s00018-024-05486-8 39520538 PMC11550308

[47] Feng S Qi Y Xiao Z Chen H Liu S Luo H CircHIPK3 relieves vascular calcification via mediating SIRT1/PGC-1α/MFN2 pathway by interacting with FUS. BMC Cardiovasc Disord. (2023) 23(1):583. 10.1186/s12872-023-03602-3 38012555 PMC10683355

[48] Badi I Mancinelli L Polizzotto A Ferri D Zeni F Burba I miR-34a promotes vascular smooth muscle cell calcification by downregulating SIRT1 (sirtuin 1) and axl (AXL receptor tyrosine kinase). Arterioscler Thromb Vasc Biol. (2018) 38(9):2079–90. 10.1161/ATVBAHA.118.311298 30026277

[49] Chen Y Huang C Zhu S-Y Zou H-C Xu C-Y Chen Y-X. Overexpression of HOTAIR attenuates pi-induced vascular calcification by inhibiting wnt/β-catenin through regulating miR-126/klotho/SIRT1 axis. Mol Cell Biochem. (2021) 476(10):3551–61. 10.1007/s11010-021-04164-8 34014438

[50] Dai X Liu S Cheng L Huang T Guo H Wang D Epigenetic upregulation of H19 and AMPK inhibition concurrently contribute to S-adenosylhomocysteine hydrolase deficiency-promoted atherosclerotic calcification. Circ Res. (2022) 130(10):1565–82. 10.1161/CIRCRESAHA.121.320251 35410483

[51] Wu C Wu Z Hu L Tang L Hu X Fang C. SIRT1/PGC-1α/Mfn2 Pathway regulates mitochondrial homeostasis in VSMC to attenuate aging-related vascular calcification. Sci Rep. (2025) 15(1):38045. 10.1038/s41598-025-21905-7 41168246 PMC12575675

[52] Liu S-M Zhang Y-R Chen Y Ji D-R Zhao J Fu S Intermedin alleviates vascular calcification in CKD through sirtuin 3-mediated inhibition of mitochondrial oxidative stress. Pharmaceuticals. (2022) 15(10):1224. 10.3390/ph15101224 36297336 PMC9608591

[53] Sun Z Zhang L Yin K Zang G Qian Y Mao X SIRT3-and FAK-mediated acetylation-phosphorylation crosstalk of NFATc1 regulates nε-carboxymethyl-lysine-induced vascular calcification in diabetes mellitus. Atherosclerosis. (2023) 377:43–59. 10.1016/j.atherosclerosis.2023.06.969 37392543

[54] Byun K-A Oh S Yang JY Lee SY Son KH Byun K. Ecklonia cava extracts decrease hypertension-related vascular calcification by modulating PGC-1α and SOD2. Biomed Pharmacother. (2022) 153:113283. 10.1016/j.biopha.2022.113283 35717781

[55] Wei W Guo X Gu L Jia J Yang M Yuan W Bone marrow mesenchymal stem cell exosomes suppress phosphate-induced aortic calcification via SIRT6–HMGB1 deacetylation. Stem Cell Res Ther. (2021) 12(1):235. 10.1186/s13287-021-02307-8 33849640 PMC8042866

[56] Tao Y Wu Y Jiang C Wang Q Geng X Chen L Saturated fatty acid promotes calcification via suppressing SIRT6 expression in vascular smooth muscle cells. J Hypertens. (2023) 41(3):393–401. 10.1097/HJH.0000000000003342 36728900

[57] Luo D Li W Xie C Yin L Su X Chen J Capsaicin attenuates arterial calcification through promoting SIRT6-mediated deacetylation and degradation of HIF-1α (hypoxic-inducible factor-1 alpha). Hypertension. (2022) 79(5):906–17. 10.1161/HYPERTENSIONAHA.121.18778 35232219

[58] Tóth A Lente G Csiki DM Balogh E Szöőr Á Nagy B Activation of PERK/eIF2α/ATF4/CHOP branch of ERS response and cooperation between HIF-1α and ATF4 promotes daprodustat-induced vascular calcification. Front Pharmacol. (2024) 15:1399248. 10.3389/fphar.2024.1399248 39144616 PMC11322142

[59] Abdelfattah AM Abdelnour HM Askar EM Abdelhamid AM Elgarhi RI. Ameliorative effect of GLP1 agonist on vascular calcification in normoglycemic aged rat aorta via miR34a/SIRT6/NRF2/HO-1 signalling pathway. Eur J Pharmacol. (2025) 1001:177741. 10.1016/j.ejphar.2025.177741 40383222

[60] Song S Yu X Xie C Li Z Zhang Y Liu Y Long-term *in vivo* administration of panaxynol alleviates diabetes-induced vascular calcification by modulating Sirt6-mediated sEH function in perivascular adipose tissue. J Agric Food Chem. (2025) 73(29):18268–79. 10.1021/acs.jafc.5c01982 40622152 PMC12291596

[61] Kensler TW Wakabayashi N Biswal S. Cell survival responses to environmental stresses via the Keap1-Nrf2-ARE pathway. Annu Rev Pharmacol Toxicol. (2007) 47(1):89–116. 10.1146/annurev.pharmtox.46.120604.141046 16968214

[62] Vigili de Kreutzenberg S Giannella A Ceolotto G Faggin E Cappellari R Mazzucato M A miR-125/Sirtuin-7 pathway drives the pro-calcific potential of myeloid cells in diabetic vascular disease. Diabetologia. (2022) 65(9):1555–68. 10.1007/s00125-022-05733-2 35708762 PMC9345831

[63] Cheng Y Zheng G Huang H Ni J Zhao Y Sun Y GLSP Mitigates vascular aging by promoting Sirt7-mediated Keap1 deacetylation and Keap1-Nrf2 dissociation. Theranostics. (2025) 15(10):4345–67. 10.7150/thno.110324 40225574 PMC11984382

[64] Ye Y Chen A Li L Liang Q Wang S Dong Q Repression of the antiporter SLC7A11/glutathione/glutathione peroxidase 4 axis drives ferroptosis of vascular smooth muscle cells to facilitate vascular calcification. Kidney Int. (2022) 102(6):1259–75. 10.1016/j.kint.2022.07.034 36063875

[65] Ma W-Q Sun X-J Zhu Y Liu N-F. Metformin attenuates hyperlipidaemia-associated vascular calcification through anti-ferroptotic effects. Free Radical Biol Med. (2021) 165:229–42. 10.1016/j.freeradbiomed.2021.01.033 33513420

[66] Wu X Li Y Zhang S Zhou X. Ferroptosis as a novel therapeutic target for cardiovascular disease. Theranostics. (2021) 11(7):3052–9. 10.7150/thno.54113 33537073 PMC7847684

[67] Ren H Shao Y Wu C Ma X Lv C Wang Q. Metformin alleviates oxidative stress and enhances autophagy in diabetic kidney disease via AMPK/SIRT1-FoxO1 pathway. Mol Cell Endocrinol. (2020) 500:110628. 10.1016/j.mce.2019.110628 31647955

[68] Peng Q Chen X Liang X Ouyang J Wang Q Ren S Metformin improves polycystic ovary syndrome in mice by inhibiting ovarian ferroptosis. Front Endocrinol (Lausanne). (2023) 14:1070264. 10.3389/fendo.2023.1070264 36755918 PMC9900736

